# Embryotoxicity assays for leached components from dental restorative materials

**DOI:** 10.1186/1477-7827-9-136

**Published:** 2011-10-06

**Authors:** Antonio Libonati, Giuseppe Marzo, Francesca G Klinger, Donatella Farini, Gianni Gallusi, Simona Tecco, Stefano Mummolo, Massimo De Felici, Vincenzo Campanella

**Affiliations:** 1 Department of Dental Science, University of Rome "Tor Vergata", Rome, Italy; 2 Department of Health Science, University of L'Aquila, L'Aquila, Italy; 3 Department of Public Health and Cell Biology, Section of Histology and Embryology, University of Rome "Tor Vergata," Rome, Italy

## Abstract

**Background:**

Currently, there are no suitable assays available to evaluate the embryotoxicity of leached components from restorative dental materials.

**Methods:**

The effect of the medium conditioned by composites and amalgam on mouse blastocysts in vitro was tested. The materials were also subcutaneously implanted, and the effect of the medium supplemented with serum from the host blood was evaluated in the embryotoxicity assay. The embryo implantation rate in the material-transplanted mothers was also evaluated.

**Results:**

The results show that while the culture in media conditioned by amalgams did not affect blastocyst development, the medium conditioned by composites caused blastocyst degeneration and apoptosis. The development of blastocysts in a medium containing serum obtained from animals after transplantation was, however, without effect. Finally, inconsistent reduction in the implantation rate in transplanted mothers was observed.

**Conclusions:**

In this study, we provide examples of in vitro and in vivo tests that may be used to evaluate embryotoxicity for dental materials. Our results show that leached components from our composite-material induced embryotoxicity in vitro, however, no toxicity was observed when subcutaneously implanted in vivo. This highlights the necessity of integrated in vitro and in vivo tests for valuable predictive estimation of embryotoxicity for complex materials.

## Background

A variety of potential toxic compounds may be released from restorative dental materials, amalgam, and composites and can diffuse into the tooth pulp or gingiva reaching the saliva and the circulating blood [1].

Dental amalgam is a mixture of mercury along with other metals, such as silver, tin, copper, and zinc. Amalgams have been used in dentistry for over 150 years because they are malleable, durable, and more affordable than gold or composites. While it is accepted that amalgam fillings release mercury, the amount of mercury released by amalgam seems negligible; thus, there is no danger from mercury leaking from fillings into the body [2]. Critics argue that long-term exposure to the low levels of mercury vapor causes neurodegenerative diseases, birth defects, and mental disorders. Although there is international agreement that the scientific data do not confirm the presence of a significant health hazard, several countries restrict the use of dental amalgams or have recommended limitations on their use.

In several European countries, dental composites are replacing amalgams as the most common restorative dental materials. Photo-cured dental composites are the most commonly placed dental restorative material. A commercial dental composite consists of a mixture of dimethacrylate monomers (resins) with up to 80% by weight of silane-coated, inorganic filler particles. The composite paste is incrementally packed into a cavity preparation, and the dentist exposes each increment for 20-40 seconds to intense visible blue light turning the paste into a durable, solid restorative material. Besides direct filling materials, resins are also used as bonding resins, like cements; dentin adhesives; and as agents for inlays, crowns, orthodontic brackets, and veneers [3]. The (co)monomers triethyleneglycoldimethacrylate (TEGDMA), hydroxyethylenemethacrylate (HEMA), urethanedimethacrylate (UDMA), and bisphenol A glycidyl methacrylate, usually abbreviated as bis-GMA, are common components of both resin and bonding components. It has been demonstrated that unconverted (co)monomers could be released from the resin composites into an adjacent aqueous phase [4]. They could be diluted by the saliva and therefore could enter the organism [5]. In 1996, Olea et al. [6] reported detectable levels of bisphenol A (BPA) in the saliva of patients treated with dental sealants, suggesting that patients receiving this treatment could be exposed to the chemical. These findings and the subsequent clinical recommendations made by the authors [6,7] stimulated public concern about this dental treatment.

Numerous cytotoxic responses to dental composites and their components have been described. For example, TEGDMA induced large deletions in the *hprt *gene of V79 cells [8]. HEMA and TEGDMA decreased the glucose formation from pyruvate in rat kidney cells [9]. With regard to reproduction, Takai et al. [10] found that BPA decreased the frequency of development of preimplantation mouse embryos, and Al-Hiyasat et al. [11] showed that intragastric administration of leached compounds from Z-100 composite or of BPA caused a significant reduction in pregnancy in mice. Strong cytotoxic effect and inhibition of cell differentiation on mouse embryonic stem (ES) cells by bis-GMA have been recently reported [12].

In the present study, in vitro and in vivo tests were performed on mouse blastocysts with the aim of evaluating embryotoxicity of leached compounds from composites and amalgam.

## Methods

All studies were approved by the local animal ethical committee, and animal care was in accordance with the institutional guidelines in compliance with national and international laws and policies (European Economic Community Council Directive 86/109, OJL 358, Dec 1, 1987 and with NIH Guide for the Care and Use of Laboratory Animals).

### Embryo collection and culture

Blastocysts were recovered on 3.5 days post coitum (dpc) (72-80 h post hCG) from the uteri of superovulated CD1 female mice mated to males of the same strain. The morning of the vaginal plug was considered as 0.5 dpc. Embryos were flushed in M2 medium with 1 mg/ml of BSA (Sigma) and cultured in groups of five in 12.5-μl drops of D-MEM containing 100 μg/ml of pyruvate, essential and nonessential amino acids, P/S (Penicillin-Streptomycin), and 10% FCS (fetal calf serum) or 20% mouse serum under mineral oil in a humidified atmosphere of 5% CO_2 _at 37°C. Blastocyst development and morphology were observed at 24 and 48 h, estimating under a stereomicroscope the degeneration, zona pellucida (ZP) hatching and the attachment of the embryos the culture dish. Moreover, the quality of the inner cell mass (ICM) and trophoblast outgrowth of the blastocyst was assessed using the well characterized assay established by Armant et al. [13].

### Dental materials

The microhybrid composite restorative resin ENAMEL plus HFO (MICERIUM, Italy) (composition: 75% silicon dioxide, 25% 1,4-Butandioldimethacrylate, Urethandimethacrylate, bis-GMA) and the dental Zn-free amalgam (IQC PALLADIUM, Germany) (composition: Hg, Cu, Sn, Ag, and Pd) were prepared as follows. Briefly, the resin was used unpolymerized or after polymerization with conventional low-power halogen curing lamp (Castellini, Bologna, Italy) for 25 sec, which is the standard time used in clinical practice; the components of amalgam were mixed for 3 h, and the dental composite was polymerized 5 min before use.

### Medium-conditioning for direct embryotoxicity assay

The specimens were prepared by condensing the materials in a glass tube with 7-mm diameter and then condensed or light-cured to obtain a dish (7 × 1 mm). The dish was then stored for 3 days in a tube with 2 ml of the cultured medium at 37°C in a continuous rotary device. After 3 days, the medium was centrifuged, filtered with 0.45 μm of Millipore filter, and immediately used for the embryotoxicity assay.

### Transplantation specimen and serum collection

Specimens prepared as reported earlier were transplanted a few minutes after preparation in adult CD1 female mice without glass tube. Mice were anaesthetized by intraperitoneal injection of avertin (0.2 ml for 10 gr); 1-mm-long incision was made on the skin of the back to insert the specimen, and the incision was then sutured by three stitches. Sham-operated animals were used as controls. After 3 days, about 0.5 ml of blood sample was taken and centrifuged (4000 rpm for 5 min) to collect the serum. The serum was heat-inactivated in water at 56°C for 40 min and added to D-MEM for the assay.

### Hoechst and TUNEL staining

Blastocysts in suspension or attached to the culture dish were fixed in formaldehyde (4% in PBS) for 10 min and washed in PBS for 15 min. Embryos were then incubated in fluorescein-conjugated dUTP ("In Situ Cell Death Detection Kit, Fluorescein" -- Roche) for 1 h at 37°C in the dark and after careful washing in PBS stained with Hoechst (5 μg/ml for 3 min). Fluorescence was observed using a Zeiss Axioplan II microscope.

### In vivo embryotoxicity

Females were transplanted with the specimen as described earlier and placed with males after 3 days from transplantation. Insemination was verified by inspection of the vaginal plug on the following early mornings. The morning of the vaginal plug was considered as 0.5 dpc. On 5.5 dpc, the females were examined for the number of embryo implantation sites by the blue Evans dye method [14,15].

### Statistics

The results were expressed as the mean ± standard error (SD). All experiments were replicated at least thrice. The means were tested for homogeneity of variance and analyzed by unpaired *t*-test. The level of significance was set at *P *< 0.05 and *P *< 0.01. Data analysis was carried out using the GraphPad Instat software.

## Results

In a first series of experiments, the effect of the medium conditioned (CM) for 3 days by polymerized or unpolymerized composites and amalgam on blastocyst development in the culture was tested. The ability of the blastocysts to hatch from the ZP and adhere to the tissue culture dish and that of the inner cell mass (ICM) and trophoblast to outgrowth over 4 days of culture was evaluated as embryonal developmental parameters.

**Figure 1:**
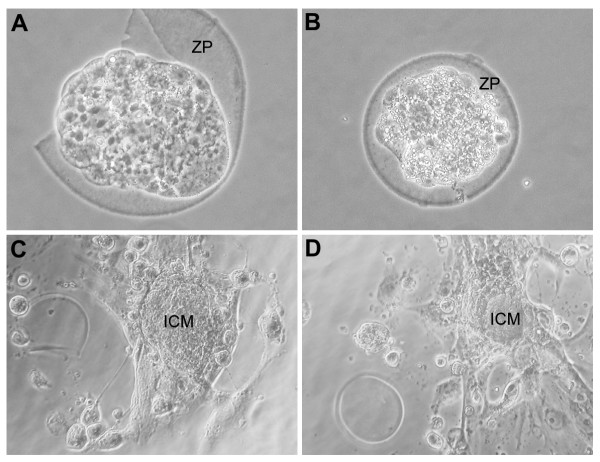
**Morphologies and development of blastocysts in culture**. Morphologies of blastocysts showing normal (A) or degenerated appearance (B) under phase contrast microscope. Note that blastocyst in A is undergoing zona pellucida (ZP) hatching. Normal inner cell mass (ICM) and trophoblast outgrowing (C) scored as +++, when compared with reduced tissue outgrowing (D) occurring in composite-conditioned medium and scored as ++. Magnification approximately 200 × (A, B) and 100 × (C, D).

The results reported in Table 1 show that the blastocysts cultured in media conditioned by amalgam underwent normal development, while 30-40% of those incubated in media conditioned by composites (C-CM), both in the polymerized and unpolymerized form, showed clear sign of degeneration (when compared with 0% of the control) when observed under a phase contrast microscope (Figures 1A and 1B). Moreover, less than 30% of viable blastocysts cultured in C-CM showed capability for ZP hatching, when compared with almost 60% of the control. Finally, ICM and trophoblast outgrowth was clearly reduced in blastocysts cultured in C-CM (Figures 1C and 1D).

**Table 1:** Effects of culture in media conditioned by composites and amalgam on blastocyst development

	Degeneration	ZP hatching	Attachment	Outgrowth
Control	0/60 (0%)	34/60 (56.7%) SD = 7,6%	34/34 (100%)	+++
Composites (polymerized)	18/57 (31.5%)** SD = 3,2%	15/39 (38,5%)* SD = 5%	14/15 (93.3%) SD = 9,8%	++
Composites (non-polymerized)	23/55 (42%)** SD = 2,1%	9/32 (28%)** SD = 2,9%	9/9 (100%)	++
Amalgam	0/45 (0%)	23/45 (51%) SD = 3,5%	23/23 (100%)	+++

*significant)ly different from the Control (t test = P < 0.05

** significantly different from the Control (t test = P < 0.001)

**Figure 2:**
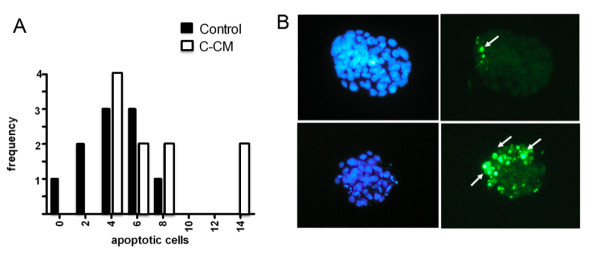
**Hoechst and TUNEL staining of blastocysts not capable of zona pellucida (ZP) hatching**. A) Frequency histogram of non-ZP hatched blastocysts with different number of apoptotic cells evaluated by the nuclear staining, Hoechst 33342, cultured in control or composite-conditioned medium (C-CM) in three independent experiments. B) Pictures of representative blastocysts cultured in control (upper) or composite-conditioned medium (below) stained with Hoechst 33342 (left) and TUNEL method (right). The arrows indicate TUNEL-positive apoptotic cells. Note a few TUNEL-positive cells in the ICM of a control blastocyst and several TUNEL-positive cells mostly concentrated in the ICM of a blastocyst cultured in C-CM. Magnification approximately 100×.

**Figure 3:**
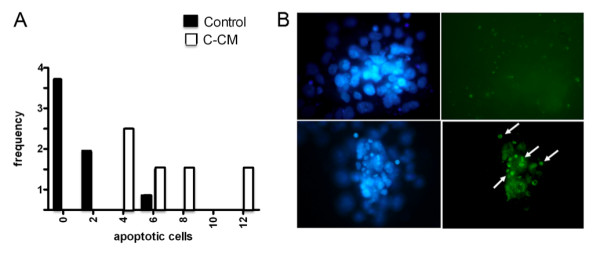
**Hoechst and TUNEL staining of blastocyst inner cell mass (ICM)**. Frequency histogram of ICM with different number of apoptotic cells evaluated by the nuclear staining, Hoechst 33342, of the embryo outgrowth cultured in control or composite-conditioned medium (C-CM) in three independent experiments. B) Pictures of representative ICM and trophoblast outgrowth cultured in control (upper) or composite-conditioned medium (below) stained with Hoechst 33342 (left) and by TUNEL method (right). The arrows indicate TUNEL-positive apoptotic cells. Note several TUNEL-positive cells in the ICM of a blastocyst cultured in C-CM. Magnification approximately 100×. All parameters were evaluated by observation under a phase contrast microscope as reported in the tests and showed in Figures 1, 2, 3. The results were obtained in at least three different experiments.

The analysis of blastocysts not capable of ZP hatching, both by the nuclear staining Hoechst 33342 and TUNEL method [14], showed increased frequency of embryos containing higher number of apoptotic cells than the control (Figures 2A and 2B). Similarly, the culture in C-CM showed increased frequency of ICM, demonstrating higher level of apoptosis (Figures 3A and 3B). However, prolonging the conditioning time up to 5 days did not increase the embryotoxicity effect (data not shown).

Using the same embryotoxicity assay, we subsequently aimed to verify whether the deleterious effects on blastocyst development observed for the leached components from dental materials were also exerted by serum obtained from the blood of animals subcutaneously implanted with the same dental materials. For this purpose, embryotoxicity assays were carried out in medium supplemented with 20% heat-inactivated serum obtained from animals after 3 days following subcutaneous implantation of composites, similar to that used in the vitro CM experiments. In such media, we found that the development of blastocysts (*n *= 30, for each tested materials) occurred at a rate similar to the control and without detectable increased level of apoptosis (data not shown).

Finally, to study whether exposure of females to the tested dental materials shortly before fertilization could influence embryo implantation in vivo, 18 females were implanted subcutaneously with dental materials 3 days before mating. With the exception of one female out of six fertilized after 1 day from mating (4 days from material implantation, see Materials and Methods), in which the number of the embryo implanted was less than half of normal (4 vs. 12+3), no reduction was observed in all other pregnant females examined (*n *= 17), which were fertilized between 1 (*n *= 5) and 5 (*n *= 12) days from mating (5-8 days from material implantation).

## Discussion

The first series of experiments carried out in vitro by culturing blastocyst in the media conditioned by dental materials suggested that compounds released from composite materials, but not from amalgam, may have direct deleterious effects on embryo development at the blastocyst stage. Interestingly, the increased frequency of blastocysts and, in particular, of ICM cells showing higher number of apoptotic cells than the control indicates that the deleterious effect by leached compounds from composites is not due to a general embryotoxicity action but rather results from a pro-apoptotic action on the ICM cell population from which the embryo proper will develop.

In line with our results, Al-Hiyasat [11] found that among a number of non-amalgam materials and some of their metabolic intermediates tested in vitro on mouse embryonic stem (ES) cells considered to have characteristics similar to ICM cells, bis-GMA caused a strong decrease in cell survival and inhibition of cell differentiation over a large range of concentrations (10-7-10-5 M) [12].

Composites contain potentially cytotoxic compounds, and numerous cytotoxic responses of dental composites and their components have been described (see Background). In the present paper, the leached embryotoxic compounds were not identified, as it was beyond the scope of the work. On the other hand, the deleterious effect of composites described here does not appear to be attributable to BPA, another major leached component from the composites [11], because up to 100 μM of BPA did not produce detectable effect on blastocyst development in the culture [[10]; data not shown].

Irrespective of the nature of the leached compounds, the lack of embryotoxicity effect found by us when the in vitro assay was carried out in medium supplemented with serum obtained from the blood of animals subcutaneously implanted with the same dental materials, suggested that such compounds might be cleared or inactivated locally or after releasing in the systemic circulation. Further experiments, including kinetic data and biochemical analyses of the sera, are needed to support such a possibility.

Similarly, we did not observed any consistent alteration in the embryo implantation rate in mothers previously implanted with dental materials.

In partial contrast with these latter results, other studies reported that leached compounds from the dental composite Z-100 or BPA administrated intragastrically daily for a total of 28 days caused a significant reduction in the number of pregnancies in the mouse [11]. However, only females exposed to 5 and 100 μg/ml/Kg of BPA showed statistically significant increase in the total number of embryo resorptions after 1 week from conception. In addition, when BPA was administrated at much higher doses of 1000 mg/Kg during the entire period of pregnancy in rats, there were increased incidences of pregnancy failure, pre- and postimplantation loss, and fetal developmental delay [16]. Different experimental protocols and species difference may explain such partial discrepancy. However, it must be pointed out that in the study carried out by Al-Hiyasat et al. [11], similar to the results of this study, no effect on the number of implanted embryos was found in females exposed to leached compounds from the Z-100 composite. Adverse effects of BPA on the fertility and reproductive system of female mice were observed at concentrations probably much higher than the dose levels expected to be leached from a single composite resin restoration [12] and were considered to result from its xenoestrogenic action on the hypothalamic-pituitary-gonadal system and modification of the uterine tissues before the arrival of the embryo [11,17-19].

## Conclusions

This study describes embryotocity in vitro and in vivo tests proposed to evidence deleterious effects of leached compounds from dental materials on early stages of embryo developmental. The in vitro tests together to other in vitro toxicity testing methods are useful for pre-screening the toxicity of materials and compounds within short time. They are presently not adequate to entirely replace animal toxicology tests. The finding that the same material does not appear to exert embryotoxic effect under the experimented in vivo conditions must be interpreted with caution. In fact, more studies are needed to identify the compounds released, the kinetics of the release under the in vitro and in vivo conditions and possibly the metabolism of the leached compounds in vivo.

## Competing interests

The authors declare that they have no competing interests.

## Authors' contributions

AL recorded the data, GM revised the text of the manuscript, FGK discussed the results, DF performed instrumental examinations, GG recorded the scientific papers on this argument, ST revised the literature review, the discussion of results and the text, and SM revised the text, and MDF and VC ideated the research and the protocol and coordinated the activities. All the authors read and approved the final manuscript.
